# Eye Size and Shape in Relation to Refractive Error in Children: A Magnetic Resonance Imaging Study

**DOI:** 10.1167/iovs.64.15.41

**Published:** 2023-12-28

**Authors:** Sander C. M. Kneepkens, Kasper Marstal, Jan-Roelof Polling, Vincent W. V. Jaddoe, Meike W. Vernooij, Dirk H. J. Poot, Caroline C. W. Klaver, J. Willem L. Tideman

**Affiliations:** 1 Department of Ophthalmology, Erasmus University Medical Center, Rotterdam, The Netherlands; 2 Department of Epidemiology, Erasmus University Medical Center, Rotterdam, The Netherlands; 3 The Generation R Study Group, Erasmus University Medical Center, Rotterdam, The Netherlands; 4 Department of Medical Informatics, Erasmus University Medical Center, Rotterdam, The Netherlands; 5 Department of Orthoptics, School of Applied Science Utrecht, Utrecht, The Netherlands; 6 Department of Radiology, Erasmus University Medical Center, Rotterdam, The Netherlands; 7 Department of Ophthalmology, Radboud University Medical Center, Nijmegen, The Netherlands; 8 Institute of Molecular and Clinical Ophthalmology, Basel, Switzerland

**Keywords:** myopia, magnetic resonance imaging (MRI), eye shape

## Abstract

**Purpose:**

The purpose of this study was to determine the association between eye shape and volume measured with magnetic resonance imaging (MRI) and optical biometry and with spherical equivalent (SE) in children.

**Methods:**

For this study, there were 3637 10-year-old children from a population-based birth-cohort study that underwent optical biometry (IOL-master 500) and T2-weighted MRI scanning (height, width, and volume). Cycloplegic refractive error was determined by automated refraction. The MRI images of the eyes were segmented using an automated algorithm combining atlas registration with voxel classification. Associations among optical biometry, anthropometry, MRI measurements, and RE were tested using Pearson correlation. Differences between refractive error groups were tested using ANOVA.

**Results:**

The mean volume of the posterior segment was 6350 (±680) mm^3^. Myopic eyes (SE ≤ −0.5 diopters [D]) had 470 mm^3^ (*P* < 0.001) and 970 mm^3^ (*P* < 0.001) larger posterior segment volume than emmetropic and hyperopic eyes (SE ≥ +2.0D), respectively. The majority of eyes (77.1%) had an oblate shape, but 47.4% of myopic eyes had a prolate shape versus 3.9% of hyperopic eyes. The correlation between SE and MRI-derived posterior segment length (*r* −0.51, *P* < 0.001) was stronger than the correlation with height (*r* −0.30, *P <* 0.001) or width of the eye (*r* −0.10, *P <* 0.001).

**Conclusions:**

In this study, eye shape at 10 years of age was predominantly oblate, even in eyes with myopia. Of all MRI measurements, posterior segment length was most prominently associated with SE. Whether eye shape predicts future myopia development or progression should be investigated in longitudinal studies.

Refractive errors affect a large part of the world population, and the prevalence of myopia, or nearsightedness, increases worldwide.^1^^–^^3^ Most myopia develops during childhood and teenage years up to adolescence, predominantly by elongation of the vitreous chamber.^4^ A proportion of the myopes will develop high myopia (≤ −6 diopters [D]), in which the axial length can grow beyond 26 mm.^5^^,^^6^ This can lead to the development of staphylomas and result in morphological changes of the optical nerve and retina and sclera with increased risk of visual impairment and blindness.^7^^–^^9^

A study among young pilots was the first to describe that those with more peripheral hyperopic defocus had more severe myopia progression.^10^ Although some reservations were expressed later about the study design and power of the evidence,^11^ animal studies confirmed this observation.^12^ This created interest in eye shape, peripheral refraction, and myopia development. Magnetic resonance imaging (MRI) can measure eye shape and determinants of eye shape independent of the eye's optics and optical power. In addition, it can measure the eye's height, width, and volume, which cannot be obtained with regular ocular biometry techniques. MRI studies in adults showed that eyes with high myopia have a prolate shape, and the eye is more curved in the posterior pole than in the periphery, in contrast to the more oblate-shaped emmetropic eyes, where the eye is more curved in the periphery than in the posterior pole.^9^^,^^13^^–^^17^ A prolate shape has been hypothesized to be a risk factor for axial eye growth, as the degree of hyperopic defocus and retinal surface area so exposed is greater, as it may contribute to the stimulus driving foveal myopia.^10^^,^^18^^–^^20^ Studies that investigated eye shape on MRI are scarce. Those available focused mainly on myopia and were performed on a relatively small set of either very young children or adults of Asian ethnicity.^13^^,^^20^^–^^26^ Automatic segmentation of the eye on MRI scans can be challenging, and recent studies have tried to solve this problem. However, these have segmented eyes with ocular pathology without reference data on healthy eyes.^27^^–^^29^ Large studies evaluating MRI-based biometry and shape for the entire spectrum of refractive errors will provide these data.

The current extensive study describes eye shape determined from MRI images, and investigates the association between shape parameters and refractive error in children.

## Materials and Methods

### General Design

This study was embedded in the Generation R Study, a population-based prospective cohort study of pregnant women and their children in Rotterdam, The Netherlands. A total of 9778 pregnant women were included in the study. All children were born between April 2002 and January 2006.^30^^,^^31^ The children were invited at ages 6 and 10 years for examination at the research center by trained nurses. Of the 9778 pregnant women, 5872 participated with their children for physical examination at the research center at 10 years of age. Of these, a total of 3637 (62%) children underwent a T2-weighted eye scan. The study protocol was approved by the Medical Ethical Committee of the Erasmus Medical Center, Rotterdam (MEC 217.595/2002/20). Written informed consent was obtained from all participants.

### Magnetic Resonance Imaging 

All participants underwent a brain MRI scan at a 3 Tesla scanner (Discovery 750, General Electric, Milwaukee, WI, USA) with an 8-channel receive-only head coil.^32^ The protocol included a 3D stabilized/variable flip angle 3D Fast spin echo T2-weighted fat suppressed scan (TR = 1440 ms, TE = 129.59 ms, field-of-view (FOV) = 256 × 256 mm, matrix size = 256 × 256; 176 sagittal slices with 1 mm, voxel size 1 × 1 × 1 mm^3^, ARC (4): phase 2.0, slice 2.0) with a scan time of 56 seconds and an echo train length of 256. Both eyes were included in the FOV of this sequence. The scans were acquired with the children in a supine position using an overhead 45 degrees inclined mirror with a fixation point at 3 meters to avoid movement artifacts. Scans were included based on visual quality inspection (author J.T.) before and after segmentation with an overlying automated segmentation raster. All images were examined for co-incidental pathologic discoveries by experienced radiographers.^33^

### Segmentation Method

**Figure 1.**
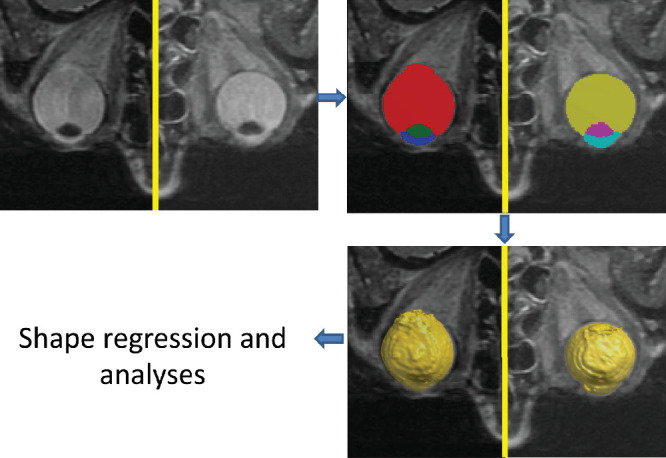
Example of segmentation, with a highly myopic eye on the left and an emmetropic eye on the right. The *left* figure shows the original acquired MRI image. The *upper right* figure depicts the separate areas that are segmented: the posterior segment in *red* and *yellow*, the lens in *green* and *purple* and the anterior chamber in *dark* *blue* and *light blue*. The *lower right* picture shows the final output of the automated segmentation.

The eyes were segmented into six regions using a combination of atlas segmentation and pixel-wise classification.^34^ First, an experienced observer (author J.T.) manually segmented 10 scans from participants with myopia, 10 scans from participants with hyperopia, and 10 scans from participants with emmetropia. These segmentations were used as atlases and to train a random forest classifier. The 30 atlases were registered to each subject image to produce a map with class probabilities for the posterior segment (PS), anterior chamber, and lens for both the left and the right eyes (Fig. 1). Then, a random forest classifier was applied to produce a second map with class probabilities. A bias was added to prevent over-ruling of segmentations inside the eyes, which might look like background. Last, the two maps were multiplied, and for each voxel the class with the maximum probability was used as the final segmentation. Table 1 shows this method's mean Dice Similarity Coefficient for 3-fold cross-validation.

**Table 1.** Results of the Three-Fold Cross-Validation of the Segmentation Method

Tissue	Dice Similarity Coefficients
Right PS	0.97 ± 0.02
Left PS	0.97 ± 0.01
Right anterior chamber	0.83 ± 0.1
Left anterior chamber	0.83 ± 0.08
Right lens	0.82 ± 0.9
Left lens	0.83 ± 0.83

For each class, we report the mean and the standard deviation of Dice similarity coefficients computed from all test images in all folds. Dice similarity of 1 is perfect overlap.

To train the classifier, segmentation labels were used as the ground truth, and the following 48 features were used to characterize each pixel: the first- and second-order derivatives, the gradient magnitude, the Laplacian, the Eigenvalues of the Hessian matrix, and the determinant of the Hessian matrix; all these features were computed on multiple scale levels of 1 mm, 1.6 mm, and 4 mm, respectively.

### Anatomic Directions

**Figure 2.**
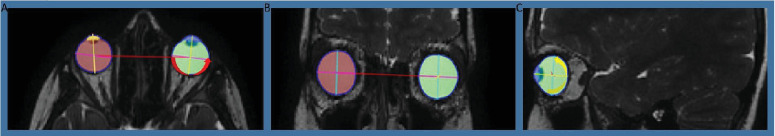
Anatomic directions are defined in an example MRI scan, with the anterior-posterior axis in *yellow*, the superior-inferior axis in *light blue*, and the left-right axis in *purple*. The *red line* connects the center of mass of the right and left eyes. The *yellow arrow* shows the vertical sphericity, and the *blue arrow* shows the horizontal sphericity.

To determine eye height and eye width, we defined an anatomic coordinate system for each eye using the segmentations in the subject image space. The eye's anterior-posterior (AP) axis was defined as the direction between the vitreous chamber's centroid and the lens's centroid. The eye's superior-inferior (SI) axis was defined as the direction orthogonal to the plane spanned by the AP axis and the direction between the centroid of the left PS and the right PS. The eye's left-right (LR) axis was defined as the direction orthogonal to the plane spanned by the AP axis and SI axis. The height of the eye was measured along the SI axis at the centroid of the PS, and the eye's width was measured along the LR axis at the centroid of the PS. The eye's length was measured along the AP axis and divided into posterior segment length and anterior chamber depth (Fig. 2).

### Shape and Volume on MRI

Sphericity (S) indicates the shape of a spheroid body when compared with that of a perfect sphere. The horizontal sphericity (hS) was calculated as height^2^/axial length^2^ −1, and the vertical sphericity (vS) was calculated as width^2^/axial length^2^-1.^35^^,^^36^ S > 0.005 was considered oblate, S < −0.005 as prolate, and −0.005<S>0.005 as spherical. The volume (mm^3^) of each region of the eye was computed from the number of segmented voxels.

### Refractive Error and Optical Biometry

Optical biometry was undertaken using the Zeiss IOL-master 500 (Carl Zeiss MEDITEC IOL-master, Jena, Germany). It included axial length, corneal radius of curvature (CR), and anterior chamber depth (ACD). For axial length, five measurements per eye were averaged to derive a mean axial length. Three keratometry measurements (K1 and K2) were taken of the right and left eyes and averaged to derive a mean CR. Axial length/CR ratio was calculated by dividing axial length (mm) by CR (mm).

The ophthalmological examination included automated cycloplegic autorefraction (Retinomax-3, Bon, Lübeck, Germany). Two drops of cyclopentolate (1%, 3 in case of dark irises), spaced 5 minutes apart, were administered at least 30 minutes before refractive error measurement in all children. No serious adverse reactions were reported. The spherical equivalent (SE) refractive error was calculated as the average sphere + 1/2 cylinder. The assumption was made that with 6 mm pupil mydriasis, complete cycloplegia was achieved as measurements were acquired in a well-lit room. Children with inadequate cycloplegia (pupil diameter <6.0 mm) were excluded from the analysis.

### Covariates

Body height of the children was measured without their shoes. Gestational age and birth weight were obtained using medical records and hospital registries. As a proxy for ethnicity, the country of birth of the mother and father was obtained by questionnaire using the method developed by Statistic Netherlands. Subsequently, these were grouped into European and non-European.^37^

### Statistical Analysis

Differences in variables between boys and girls or the three groups (hyperopia ≥ +2.0 D, emmetropia < +2.0 ≥ −0.5 D, and myopia ≤ −0.5 D) were tested using chi-square and ANOVA tests. Correlation among the MRI variables (height, width, PS depth, PS volume, lens volume, anterior chamber volume, and prolateness) and ocular measurements (SE, axial length/CR, axial length, CR, anterior chamber depth, and axial length growth) or gestational age and anthropometry (birth weight and body height) were tested with Pearson correlation. The association between spherical equivalent and horizontal shape was determined using linear ordinary least squares regression models, with restricted cubic splines with three knots (the 10th, 50th, and 90th percentiles). The associations between axial length, height, or width of the eye with SE were tested using linear regression models adjusted for age and gender. Additionally, a Bland-Altman plot with an indication of limits of agreement was used to compare axial length measured using optical biometry and that measured with MRI. Statistical tests were performed using SPSS (version 21.0.0.0) and R (version 4.2.1).

## Results

In total, 3637 children underwent MRI scans, and 2963 of 3637 (81.5%) children were included in the analyses. Of those excluded, 523 (14.4%) children had low-quality MRI scans (motion artifacts 441, braces 56, and incorrect positioning of the participant 26). Examples of a good quality and bad quality scans can be found in the supplements (Supplementary Figs. S1–S3). Optical biometry had not been performed in 151 (4.2%) of the children. The excluded children did not show significant differences from the included children in axial length (*P* = 0.28), axial length/CR ratio (*P* = 0.56), or SE (*P* = 0.32). The children were, on average, 10.1 (0.6) years of age; and 51.5% (1525) were girls. The characteristics of the cohort, ocular biometry, and volume measurements are summarized in Table 2. Cycloplegic refractive error data was available for 1699 children (57.5%). Of these, 209 (12.3%) were myopic and 127 (7.5%) were hyperopic.

**Table 2.** General and Ocular Characteristics From 10-Year-Old Boys and Girls From the Generation R Study

	All *N* = 2963	Boys *N* = 1438	Girls *N* = 1525	*P* Value^‡^
**General measurements**				
Age child (y)	10.1 (0.59)	10.2 (0.61)	10.1 (0.56)	0.002
European ethnicity (%)^†^	70.0 (2074)	69.0 (990)	71.0 (1084)	0.13
Body height (cm)	141.7 (6.5)	141.8 (6.3)	141.6 (6.7)	0.48
Birthweight (grams)	3434 (564)	3521(555)	3352 (561)	<0.001
Gestational age (wk)	39.8 (1.8)	39.9 (1.8)	39.7 (1.9)	0.006
**Optical biometry and auto refractor**				
Axial length (mm)	23.11 (0.83)	22.86 (0.77)	23.39 (0.80)	<0.001
Axial length growth (mm/y)	0.21 (0.09)	0.21 (0.09)	0.21 (0.08)	0.69
Corneal radius (mm)	7.78 (0.26)	7.85 (0.25)	7.72 (0.24)	<0.001
AL/CR ratio	2.97 (0.09)	2.98 (0.10)	2.96 (0.09)	<0.001
Spherical equivalent (D)^§^	0.74 (1.30)	0.68 (1.28)	0.72 (1.31)	0.40
**MRI measurements**				
Posterior segment length (mm)	17.02 (0.80)	17.25 (0.80)	16.80 (0.74)	<0.001
Posterior segment height (mm)	23.57 (0.95)	23.72 (0.95)	23.43 (0.93)	<0.001
Posterior segment width (mm)	23.73 (0.95)	23.97 (0.93)	23.51 (0.91)	<0.001
Posterior segment volume (mm^3^)	6350 (680)	6530 (680)	6180 (630)	<0.001
Lens volume (mm^3^)	84 (13)	85 (13)	84 (13)	0.03
Anterior chamber volume (mm^3^)	240 (35)	240 (36)	230 (32)	<0.001
**Optical biometry and MRI**				
Vertical prolateness	0.042 (0.068)	0.031 (0.067)	0.052 (0.066)	<0.001
Horizontal prolateness	0.056 (0.066)	0.053 (0.067)	0.059 (0.065)	0.005
Oblate eye shape (%)^†^^,^^*^	78.6 (2328)	77.6 (1116)	79.5 (1212)	0.01
Spherical eye shape (%)^†^^,^^*^	4.5 (133)	3.7 (53)	5.2 (80)	–
Prolate eye shape (%)^†^^,^^*^	16.9 (502)	18.7 (269)	15.3 (233)	–

AL, axial length; CR, corneal radius of curvature; SE, spherical equivalent, except where indicated otherwise.

All data are presented as the mean (SD).

* Shape was the horizontal eye shape.

† Data are presented as % (*N*).

‡ *P* values were calculated using the Student's *t*-test or the chi-square test.

§ Children with cycloplegic refractive error, MRI data and axial length, *N* = 1699 (822 boys and 877 girls).

### Optical Biometry and MRI

**Figure 3.**
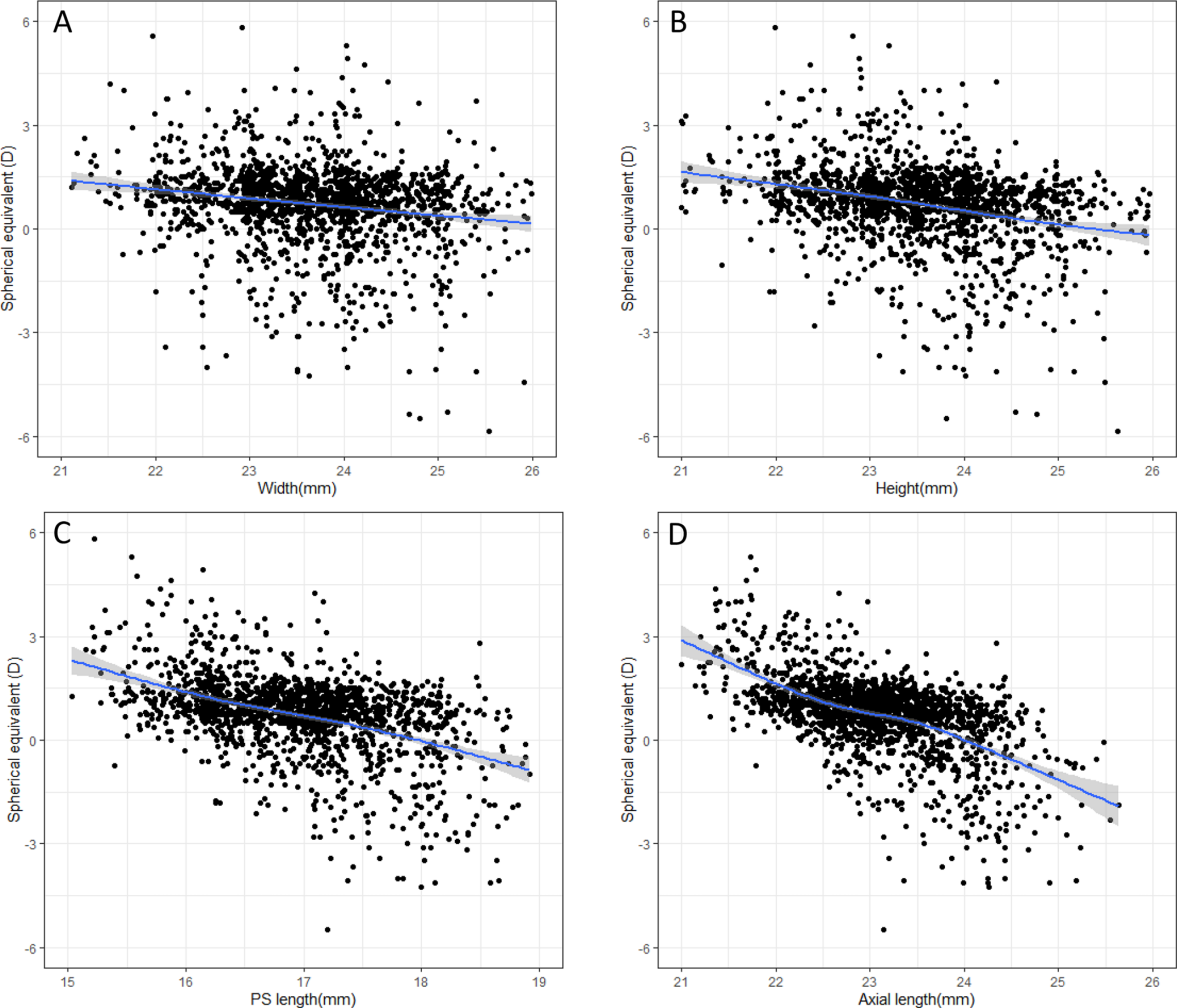
Spherical equivalent (D) as a function of width (**A**), height (**B**), and length (**C**), of the posterior segment and spherical equivalent as a function of the axial length (**D**) of the eye. The 95% confidence intervals are depicted as a *shaded line* around the *blue line*.

The mean axial length measured using optical biometry was 23.11 (0.83) mm, differing on average 0.18 mm (95% confidence interval around the mean difference −1.07 to 0.71 mm) from axial length measured using MRI (Supplementary Fig. S4). These axial length measurements were highly correlated (*r* = 0.87, *P <*0.001). Axial length measured on MRI and axial length measured with optical biometry were both correlated with spherical equivalent (*r* = 0.52, *P* < 0.001 vs. 0.61; *P* < 0.001). The correlation between the axial length measured by optical biometry and the height and width of the eye measured by MRI was 0.63 (*P* < 0.001) and 0.66 (*P* < 0.001), respectively. Axial length and ACD measured using optical biometry showed the highest correlation with PS length, followed by height, and the least with the eye width (Table 3, Fig. 3).

**Table 3.** Correlation Between Ocular Measures Obtained With Optical Biometry and Ocular Biometric Parameters Measured on MRI

	Pearson Correlation Coefficient of MRI Measurements of the Eye							
	Height	Width	PS Depth	PS Volume	Lens Volume	AC Volume	Vertical Shape	Horizontal Shape
**Ocular measurements**								
SE (D)	−0.303^**^	−0.222^**^	−0.507^**^	−0.383^**^	−0.115^**^	−0.226^**^	0.266^**^	0.396^**^
AL/CR	0.204^**^	0.100^**^	0.413^**^	0.265^**^	0.086^**^	0.224^**^	−0.326^**^	−0.477^**^
AL (mm)	0.627^**^	0.656^**^	0.868^**^	0.807^**^	0.286^**^	0.475^**^	−0.307^**^	−0.297^**^
CR (mm)	0.499^**^	0.629^**^	0.571^**^	0.639^**^	0.232^**^	0.328^**^	−0.024	−0.133^**^
ACD (mm)	0.150^**^	0.071^**^	0.203^**^	0.164^**^	0.133^**^	0.335^**^	−0.255^**^	−0.367^**^
AL growth (mm/y)	0.232^**^	0.185^**^	0.442^**^	0.321^**^	0.100^**^	0.205^**^	−0.275^**^	−0.347^**^
**Other measurements**								
Body height (cm)	0.253^**^	0.248^**^	0.180^**^	0.261^**^	0.055^**^	0.186^**^	0.115^**^	0.107^**^
Birthweight (kg)	0.151^**^	0.183^**^	0.141^**^	0.190^**^	0.042^*^	0.137^**^	0.013	0.052^**^
Gestational age (wk)	0.047^*^	0.042^*^	0.021	0.051^**^	0.011	0.032	0.019	0.012

AC, anterior chamber; ACD, anterior chamber depth. AL, axial length; CR, corneal radius of curvature; PS, posterior segment; SE, spherical equivalent.

Correlations were tested using Pearson correlation.

* *P* < 0.05.

** *P* <0.01.

On MRI, the eyes had a larger width than height (mean = 23.73 [SD = 0.94] vs. 23.57 [0.95] mm), the mean axial length measured on MRI was 22.94 (0.94) mm, and the mean PS length was 17.02 (0.80) mm. The mean total volume of the eyes was 6670 mm^3^ with a PS volume of 6350 mm^3^. Boys had a longer, higher, and wider PS than girls with a higher volume of PS, lens, and anterior chamber (lens volume *P =* 0.03, all others *P* < 0.001; see Table 2). The correlation between PS height and width was 0.67 (*P* < 0.001).

Hyperopic eyes had a volume of 6370 mm^3^, emmetropic eyes of 6610 mm^3^, and myopic eyes had the largest volume of 7240 mm^3^ (*P* < 0.001). The difference between the myopic and hyperopic eyes was lowest for width (0.7 mm), whereas the height difference was 1.2 mm, and the PS length difference was 1.5 mm (Table 4). The correlation between SE and axial length was r −0.61, between SE and height of the eye r −0.31, and between SE and width of the eye (*r* −0.22; see Fig. 3).

**Table 4.** Ocular Biometry Measured on MRI and Optical Biometry in Relation to Refractive Error

	Refractive Error Category			
	Hyperopia	Emmetropia	Myopia	*P* Value
**MRI measurements**				
Height	22.9 (0.8)	23.5 (0.9)	24.1 (0.9)	<0.001
Width	23.3 (0.9)	23.7 (0.9)	24.0 (1.0)	<0.001
PS length	16.2 (0.6)	16.9 (0.7)	17.7 (0.9)	<0.001
PS volume	5.80 (0.5)	6.30 (0.6)	6.77 (0.8)	<0.001
Lens volume	0.081 (0.01)	0.084 (0.01)	0.088 (0.01)	<0.001
AC volume	0.22 (0.03)	0.24 (0.03)	0.25 (0.04)	<0.001
Vertical shape	0.077 (0.06)	0.041 (0.06)	0.008 (0.06)	<0.001
Horizontal shape	0.113 (0.08)	0.058 (0.06)	0.005 (0.06)	<0.001
Oblate shape	87.5	71.5	53.1	<0.001
Sphere shape	0.8	4.8	1.9	<0.001
Prolate shape	11.7	23.6	45.0	<0.001
**Optical biometry**				
Axial length	22.04 (0.6)	23.07 (0.7)	23.98 (0.83)	<0.001
Corneal radius (mm)	7.76 (0.25)	7.79 (0.25)	7.72 (0.25)	0.002
AL/CR ratio	2.84 (0.07)	2.96 (0.06)	3.11 (0.09)	<0.001

AC, anterior chamber; PS, posterior segment.

Average (SD) of the ocular biometry measurements per refractive error category.

*P* values were calculated using ANOVA or chi square test.

### Eye Shape

**Figure 4.**
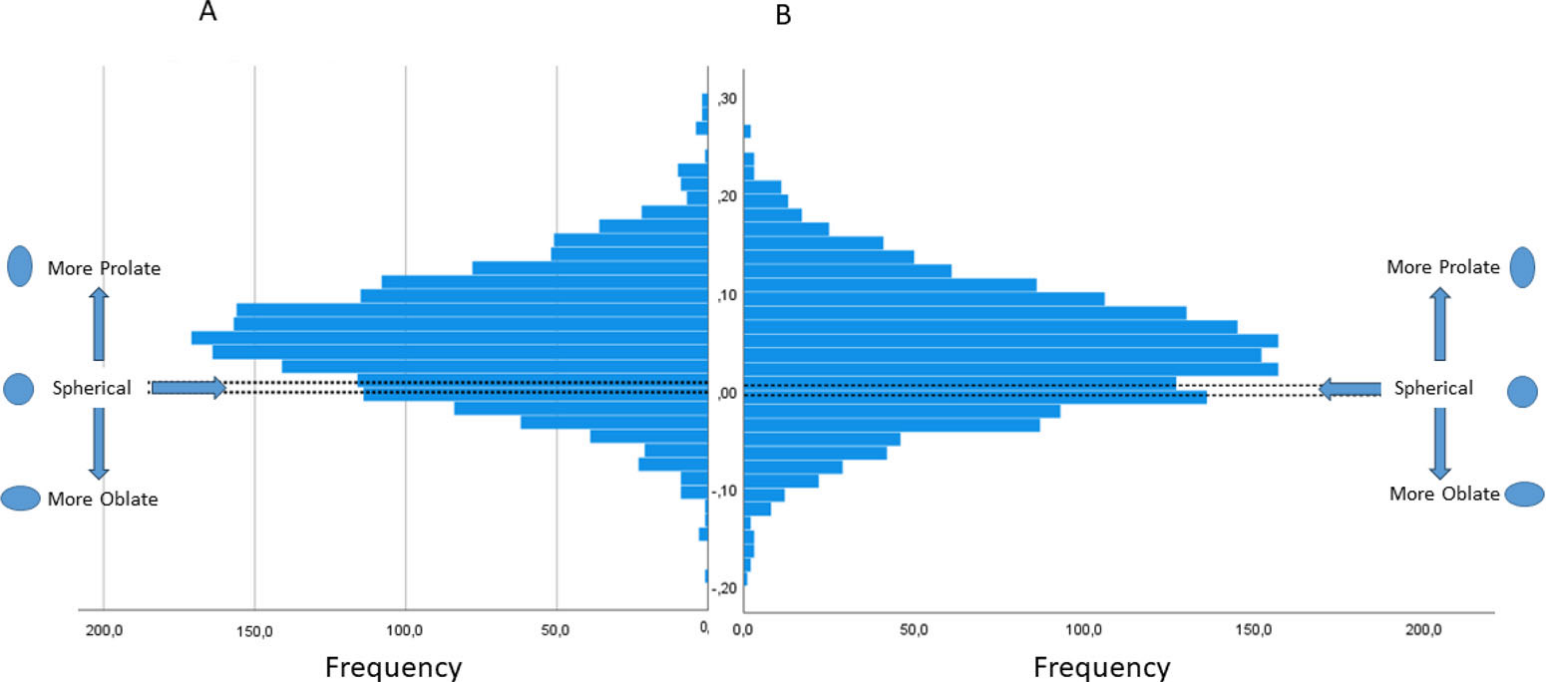
The distribution of horizontal eye shape (**A**) and vertical eye shape (**B**). A spherical shape (−0.005<S>0.005) is represented between the *dotted lines*.

The mean horizontal eye shape was 0.056 (0.068), and the mean vertical eye shape was 0.042 (0.066). The proportion of horizontal oblates was 78.6%, of spheres 4.5% (shape = 0 +/−0.005), and of prolates 16.9% (Fig. 4). Of the myopic participants, 47.4% (*n* = 99) had a prolate shape; of the hyperopic participants, 3.9% (*n* = 5) had a prolate shape.

**Figure 5.**
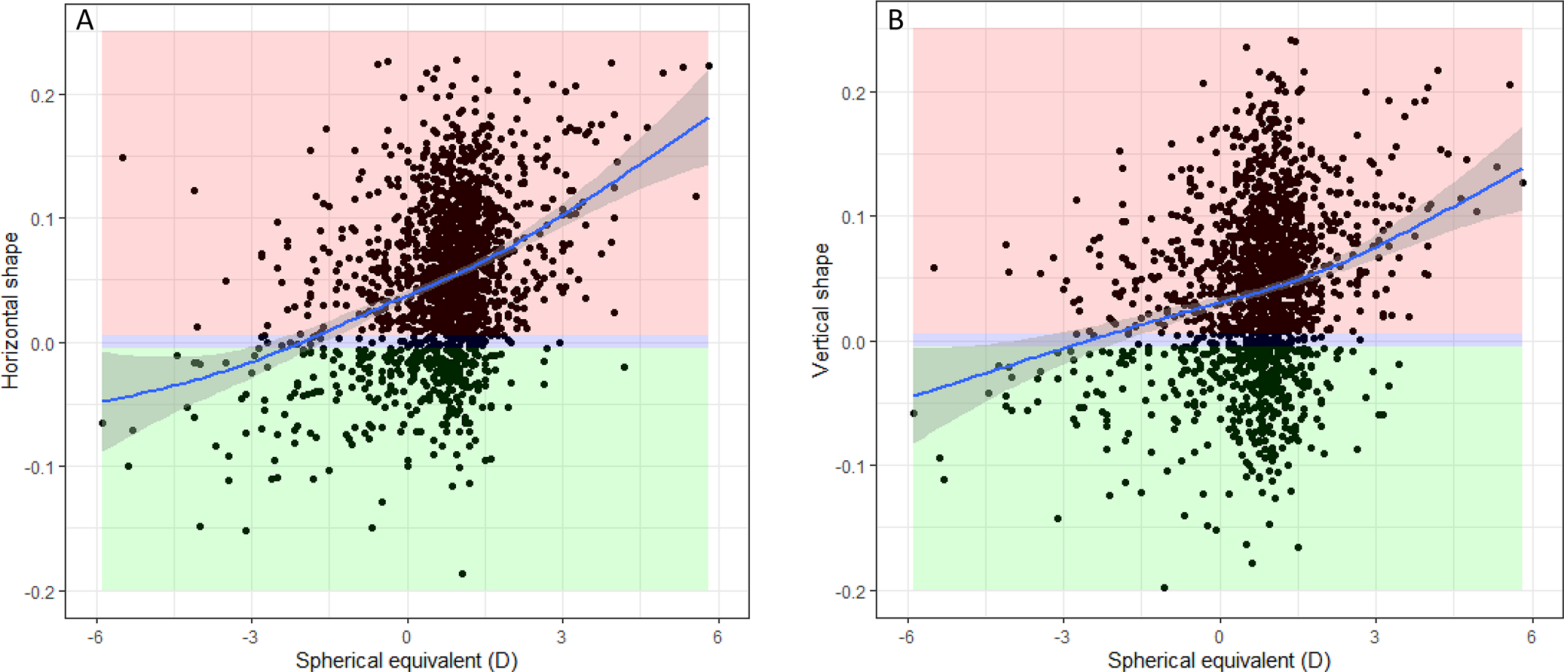
The association between spherical equivalent and horizontal eye shape (**A**) and vertical eye shape (**B**). A spherical shape (−0.005<S>0.005) is represented in the *blue-colored area*, an oblate shape (S > 0.005) in the *red-colored area*, and a prolate shape (S < −0.005) in the *green-colored area*. The 95% confidence intervals are depicted as a *shaded line* around the *blue line*.

The proportion of vertical oblates was 71.4%, of spheres 4.4%, and of prolates 24.2%. Of the myopic participants, 45% (*N* = 94) had a prolate shape; of the hyperopic participants, 11.8% (*N* = 15) had a prolate shape. For both vertical and horizontal shape, myopic refractive errors were more often observed in eyes with a prolate shape (Fig. 5). In 72% of the cases, the vertical shape was the same as the horizontal shape.

### Birth Parameters

Body height, birth weight, and gestational age had a higher correlation with the height (0.253, 0.151, and 0.047, respectively) and width of the eye (0.248, 0.183, and 0.042, respectively) than with the PS length (0.180, 0.141, and 0.021, respectively; see Table 3). Age, gender, body height, body weight, and birth weight together explained 16% of the variance in PS volume, 15% of the variance in width, 13% of the variance in height of the eye, and 13% of the PS length.

## Discussion

This is the first European study to provide normative data on ocular shape in a large population-based group of school-aged children presenting with a wide spectrum of refractive errors. We found that most children had an oblate eye shape. Hyperopic and emmetropic eyes were predominantly oblate shaped; this was also true for most myopic eyes except for those with higher refractive errors and longer axial lengths. Posterior segment length had the highest association with refractive error, but height and width also increased with more myopic refractive error. Aside from a relation with myopia, eye volume was associated with age, male gender, and birth weight. The width of the eye was related to body height as well as birth weight.

The study of ocular biometry on MRI images has several advantages, such as the possibility of studying the dimensions of the eye in all directions as well as volumes. A few previous studies reported eye measurements on MRI in babies and adults. Newborns were reported to have an average eye volume of 2428 mm^3^, whereas our 10-year-old children had an average eye volume of 6670 mm^3^.^15^ This suggests a volume increase by a factor of 2.75 in the first decade. Ethnic differences may play a role, as children of the Singapore STARS study already had an eye volume of 6690 mm^3^ at the age of 6.5 years. This is similar to our 10-year-old children and corresponds to the same mean spherical equivalent (0.70 D vs. 0.65 D).^17^ Our mean posterior segment length was only slightly shorter than that found in a study of adults, but this population consisted of predominantly female subjects who are known to have shorter axial lengths.^16^ With higher myopic refractions, axial length increased by a factor of 3 compared to the width of the eye and by 1.5 compared to the height of the eye. This is in line with measurements that have been reported before.^13^ Another advantage of using MRI imaging is the possibility of assessing the shape of the eye in a three-dimensional plane without it being affected by the eye's optical power. We found that the majority of our participants had an oblate eye shape; 47.4% of myopic participants had a prolate eye shape. Prolateness increases with a higher axial length, and the prolateness is more in the vertical plane than in the horizontal plane. Several studies using various methods of imaging describe that the eye becomes more prolate with increasing myopia in both children and adults.^15^^,^^20^^,^^25^^,^^38^ The eyes of our participants were not fully grown yet and, with age, will increase more in axial length than in height and in width. In addition, the level of myopia at which the prolate change becomes most obvious is scarce at the age of 9 years.^36^ Peripheral refraction studies had similar results with more hyperopic defocus in the periphery in myopic eyes, indicating a more prolate shape, and more myopic defocus in hyperopic eyes, indicating a more oblate shape.^39^^,^^40^ Some studies identified additional asymmetry in the peripheral refraction in the horizontal and vertical axis.^41^ In future studies, we will use our segmentations to create personal peripheral refraction profiles for our participants using ray-tracing models.^42^ This will allow us to study the relationship among peripheral refraction, eye shape, and myopia progression.^43^^,^^44^

Our study also has some limitations, such as the cross-sectional design of the analysis, the relatively low number of children with extreme refractive errors, and the limited quality of MRI scans in 15% of our population. MRI scanning is a fairly difficult examination for children at this age and sensitive to braces wear. Dropout seemed unbiased as axial length, corneal radius, and refractive error were similar in children who were in or excluded from analyses. Involuntary eye movement can also cause poor image quality and is very difficult to control. Vacuum polystyrene pads or higher-resolution scanners could mitigate this problem but were not available in this study.^56^^–^^58^ We used a head coil and fixation point to counteract unintended movements as well as possible. In addition, the different structures of the eye have different intensities on MRI images, which can hamper measuring exact anatomical structures. On T2 weighted MRI scans, the cornea and sclera are visible as hypo-intense layers relative to their surrounding structures. Therefore, our method of automatic segmentation does not include the thickness of these structures.^45^ Axial length measured using optical biometry measures from the anterior corneal surface to the retinal pigment epithelium. Therefore, we expected the axial length measured by MRI to slightly underestimate the axial length.^46^ Next to that, the spatial resolution of our segmentation method uses a resolution of 1 × 1 × 1 mm^3^ per voxel, which is less precise than the more modern techniques (0.5 × 0.5 × 1.0 mm^3^).^44^ The scan protocols were designed in 2010; scanning time was restricted to fit into the busy study schedule. Nevertheless, our large study sample enables the detection of trends despite the relative imprecision. The mean difference in axial length between MRI and IOL master we found is similar to the difference found in the literature (−0.18, 95% confidence interval [CI] = −1.07 to 0.71) mm; however, these studies reported tighter limits of agreement due to their higher image resolution^44^^,^^47^ (see Supplementary Fig. S4). Axial length differences in the literature between MRI and optical biometry measurements are between −0.1 and −0.8 mm, depending on the software used.^47^ More advanced automatic segmentation can reach a mean absolute difference of −0.1 mm.^44^

The strengths of this study are the population-based setting, the three-dimensional biometric eye measurements based on MRI images, and the large study population of homogeneous age encompassing the whole spectrum of cycloplegic refractive error and early life growth data. Considering that the highest incidence of myopia in Western European children is between 10 and 15 years of age, follow-up measurements of eye shape as the children grow older will provide further insights into the consequences of eye shape.^8^

Gaurisankar et al. report a strong correlation between axial length and myopic refraction and a somewhat weaker correlation between ACD and refractive error in their meta-analysis. They suggest an increase in axial length and ACD will lead to a more myopic refractive error.^48^^,^^49^ These findings are in line with the findings in our study (see Table 3). We find a strong correlation between PS length and axial length measured on MRI and refractive error, suggesting a longer axial length leads to a more myopic refractive error. We also find a smaller correlation between the ACD and refractive error, suggesting a deeper anterior chamber is associated with a more myopic refractive error. However, the most important determinant of SE is the PS length. Various anthropometric measurements (i.e. body height and body weight) have been associated with the eye's axial length in young children and adults.^50^^–^^52^ Our study shows that the width and height of the eye appear to be more correlated with birth weight and body height than axial length, and these measures are less prone to change when myopic refractive error increases. The relatively larger axial length growth with increasing myopia may result in a more curved posterior pole and a prolate shape in myopic eyes. This may increase peripheral hyperopic defocus, which can trigger further axial elongation.^10^^,^^19^ Peripheral hyperopic defocus is currently already a target for treatment by the use of multifocal soft contact lenses, orthokeratology (ortho-K), and DIMS and H.A.L.T. technology spectacle lenses.^53^^–^^55^ Effectivity of these therapies is found to be dependent of the peripheral shape at baseline.^54^ Suggesting there is a need for more personalized therapy.

In conclusion, our study showed that the eyes of 10-year-old children in Rotterdam are mainly oblate shaped, even in those with myopia. In this population-based study, a continuous scale of eye volume, height, width, and shape were strongly related to refractive error. Eye dimensions were correlated with height and birth weight, with the highest correlation for the eye's width. Future longitudinal studies are needed to determine whether eye size and shape can predict the rate of eye growth and myopia progression.

## Supplementary Material

Supplement 1
