# Supplementary material for: Eye Size and Shape in Relation to Refractive Error in Children: A Magnetic Resonance Imaging Study
Source: Invest Ophthalmol Vis Sci. 2023 Dec 28;64(15):41. doi: 10.1167/iovs.64.15.41 (PMC10756250; doi:10.1167/iovs.64.15.41)
Supplement: Supplement 1 [file iovs-64-15-41_s001.pdf]

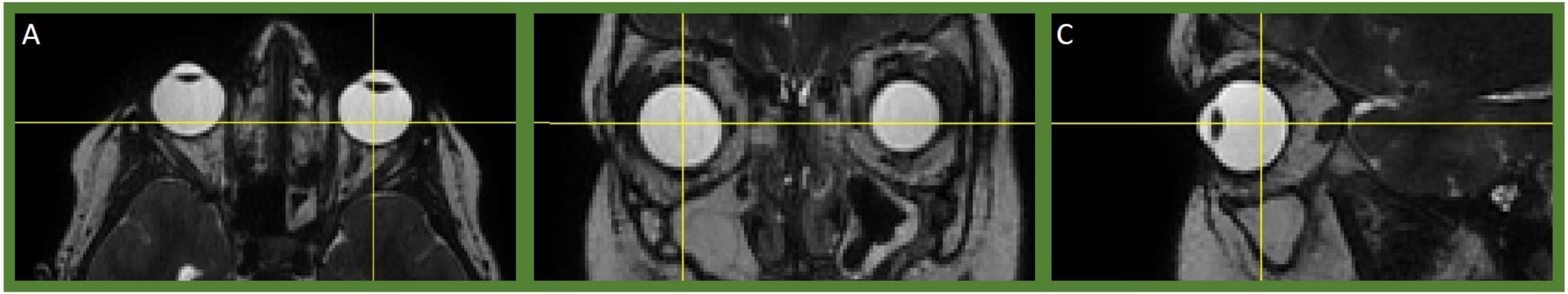

Supplementary figure 1: example of a good quality MRI scan.

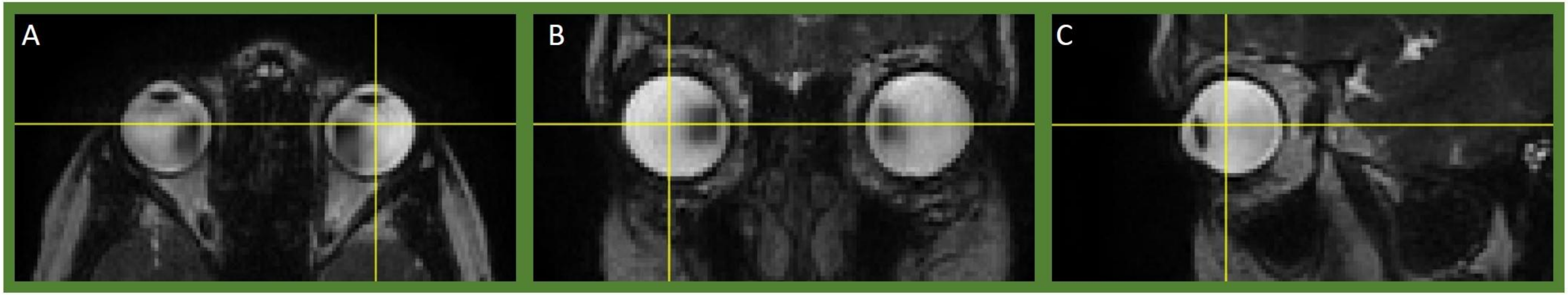

Supplementary figure 2: example of an excluded MRI scan with artifact caused by braces.

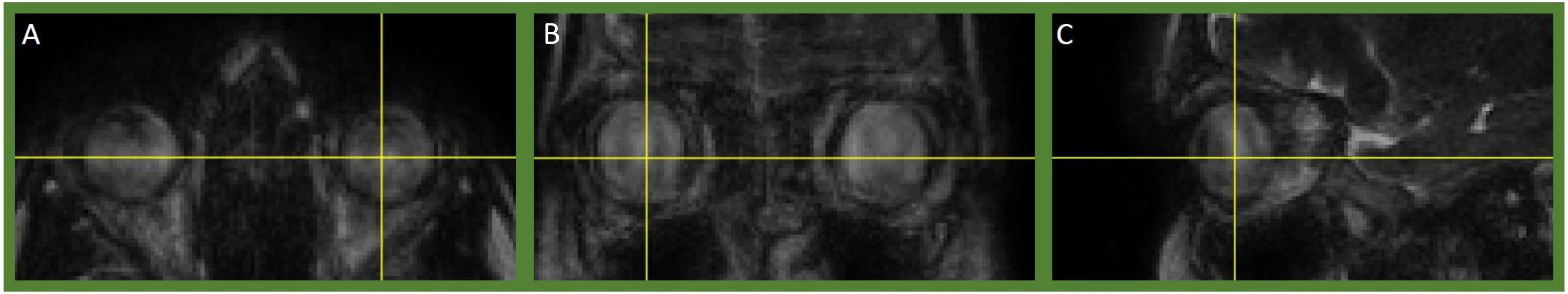

Supplementary figure 3: example of excluded MRI scan due to movement artifact.

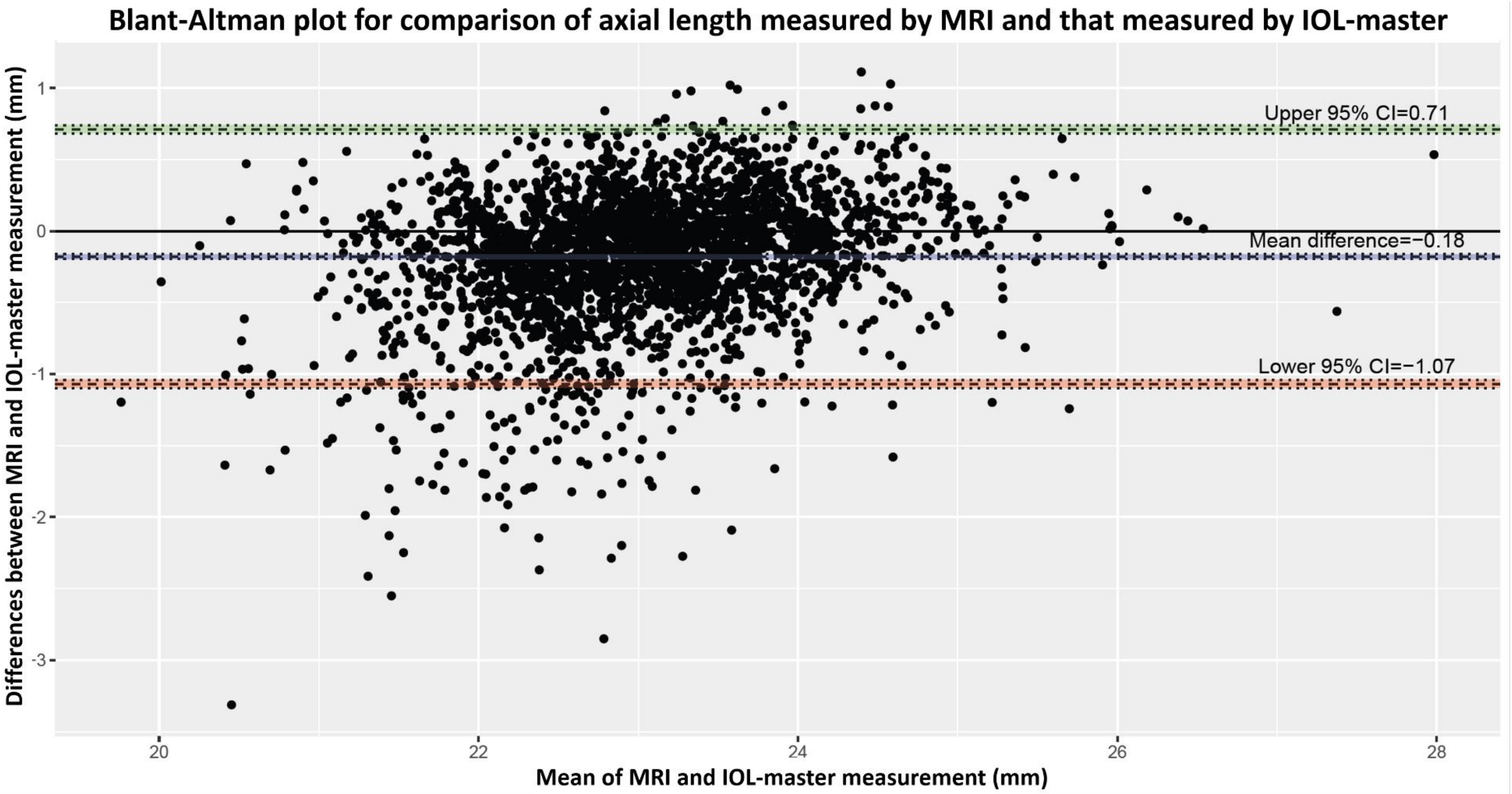

Supplementary figure 4: Bland-Altman plot for comparison of axial length measured by MRI and that measured by using optical biometry. Mean difference is indicated by the blue line, lower 95% confidence intervals is indicated by the red line and upper 95% confidence interval as indicated by the green line.
